# The Role of Mitochondrial H^+^-ATP Synthase in Cancer

**DOI:** 10.3389/fonc.2018.00053

**Published:** 2018-03-07

**Authors:** Pau B. Esparza-Moltó, José M. Cuezva

**Affiliations:** ^1^Departamento de Biología Molecular, Centro de Biología Molecular Severo Ochoa (CSIC-UAM), Centro de Investigación Biomédica en Red de Enfermedades Raras CIBERER-ISCIII, Instituto de Investigación Hospital 12 de Octubre (i+12), Universidad Autónoma de Madrid, Madrid, Spain

**Keywords:** oxidative phosphorylation, ATPase inhibitory factor 1, mitohormesis, metabolic reprogramming, hepatocarcinogenesis, inflammation

## Abstract

Cancer cells reprogram energy metabolism by boosting aerobic glycolysis as a main pathway for the provision of metabolic energy and of precursors for anabolic purposes. Accordingly, the relative expression of the catalytic subunit of the mitochondrial H^+^-ATP synthase—the core hub of oxidative phosphorylation—is downregulated in human carcinomas when compared with its expression in normal tissues. Moreover, some prevalent carcinomas also upregulate the ATPase inhibitory factor 1 (IF1), which is the physiological inhibitor of the H^+^-ATP synthase. IF1 overexpression, both in cells in culture and in tissue-specific mouse models, is sufficient to reprogram energy metabolism to an enhanced glycolysis by limiting ATP production by the H^+^-ATP synthase. Furthermore, the IF1-mediated inhibition of the H^+^-ATP synthase promotes the production of mitochondrial ROS (mtROS). mtROS modulate signaling pathways favoring cellular proliferation and invasion, the activation of antioxidant defenses, resistance to cell death, and modulation of the tissue immune response, favoring the acquisition of several cancer traits. Consistently, IF1 expression is an independent marker of cancer prognosis. By contrast, inhibition of the H^+^-ATP synthase by α-ketoglutarate and the oncometabolite 2-hydroxyglutarate, reduces mTOR signaling, suppresses cancer cell growth, and contributes to lifespan extension in several model organisms. Hence, the H^+^-ATP synthase appears as a conserved hub in mitochondria-to-nucleus signaling controlling cell fate. Unraveling the molecular mechanisms responsible for IF1 upregulation in cancer and the signaling cascades that are modulated by the H^+^-ATP synthase are of utmost interest to decipher the metabolic and redox circuits contributing to cancer origin and progression.

## Overview of Metabolic Reprogramming in Cancer

**Figure 1:**
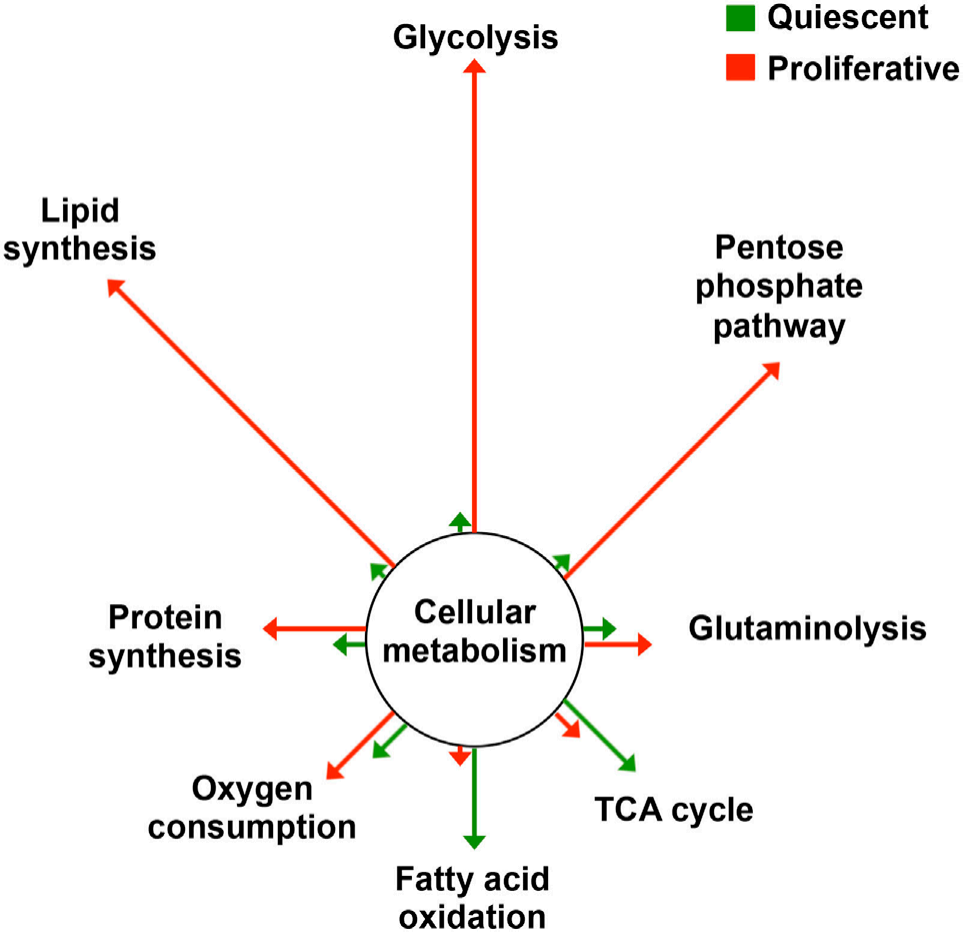
Metabolic reprogramming in proliferation. The changes in flux through the main pathways of cellular metabolism are depicted in proliferative cells (red) relative to that of quiescent cells (green). Arrows’ length is proportional to the relative change in flux. Data adapted from Ref. (2–5). Abbreviation: TCA cycle, tricarboxylic acid cycle.

Cancer cells experience a series of alterations during oncogenic transformation that confer them new features (1). Cellular metabolism is a central player in the acquisition of this new phenotype. Indeed, cancer cells are highly proliferative and readapt their metabolism to meet the demands imposed by the new phenotype, namely higher requirements of metabolic energy and of precursors for biosynthetic purposes (Figure 1) (2–5). One prominent feature of the metabolic reprogramming experienced by cancer cells is an enhanced glycolytic rate in the presence of oxygen, what is known as aerobic glycolysis (6) (Figure 1). Glycolysis provides cancer cells with various metabolic precursors that serve for the synthesis of amino acids, nucleotides, and lipids, as well as reducing power and ATP. In addition, mitochondrial function is readapted during oncogenic transformation and mutations in genes encoding mitochondrial proteins contribute to cancer development (7, 8). In particular, the relative contribution of mitochondria to energy provision is reduced, the organelles becoming mostly dedicated to produce anabolic precursors through the tricarboxylic acid (TCA) cycle (9) (Figure 1). Likewise, mitochondria, which are crucial hubs in intracellular signaling (10), readapt this function in cancer (11). Intermediates of the TCA cycle contribute to signaling tumorigenesis (12) and mtROS, which are key mediators in mitochondrial communication (13), activate signaling pathways that promote cell proliferation and tumorigenesis (14, 15). Moreover, mitochondrial dynamics is also reprogrammed in cancer (16).

## The H^+^-ATP Synthase is Downregulated in Cancer

A critical enzyme complex within the mitochondria is the H^+^-ATP synthase, the rotatory engine of the inner mitochondrial membrane responsible for ATP synthesis by oxidative phosphorylation (OXPHOS) (17). The H^+^-ATP synthase consumes the proton electrochemical gradient generated across the inner mitochondrial membrane by the electron transport chain to drive ATP synthesis (17, 18). In addition, the H^+^-ATP synthase is a critical component of the mitochondrial permeability transition pore (PTP) whose prolonged opening triggers the execution of cell death (19–21). Although its mechanism of participation in PTP opening is currently debated (22–24), recent findings have mapped the residues in subunits of the H^+^-ATP synthase for Ca^2+^ activation (25) and pH inhibition (26) of the PTP, reinforcing the role of the H^+^-ATP synthase in PTP function and providing first evidence that single point mutations in the enzyme affect PTP modulation. Hence, the H^+^-ATP synthase integrates the bioenergetic and death-signaling functions of mitochondria, what makes it a relevant target for oncogenic transformation (27, 28). Actually, mutations in the mitochondrial-encoded subunit *a* of the H^+^-ATP synthase (MT-ATP6), which are found in different human carcinomas, promote tumor growth by restraining cell death (29, 30). Recent findings in yeast MT-ATP6 mutants have confirmed the role of these mutations in the PTP response to Ca^2+^ (31), providing additional genetic evidence that supports the involvement of mutations in the H^+^-ATP synthase in PTP functioning during carcinogenesis. However, it should be noted that the two mutations in MT-ATP6 impact the PTP response only when the function of the outer mitochondrial membrane porin complex is perturbed (i.e., OM45-GFP background) (31). In fact, permeability transition has been documented in rho0 cells that lack mtDNA (32), highlighting the relevance of the genetic background of the cancer cell for the desensitization of the PTP.

Regardless of oncogenic mutations on the H^+^-ATP synthase, it has been documented that the relative expression of the catalytic subunit of the complex (β-F1-ATPase) is downregulated in most prevalent human carcinomas when compared with the corresponding normal tissues (33, 34) [for review, see Ref. (2)]. The relative expression of β-F1-ATPase in the tissue provides a “bioenergetic signature” of the carcinoma that informs of the overall capacity of mitochondria. The bioenergetic signature [also known as the bioenergetic cellular (BEC) index (2)], is assessed as the protein ratio between β-F1-ATPase and GAPDH and has been shown to be significantly reduced in colon, lung, breast, gastric, and renal carcinomas (2, 33). Interestingly, the quantification of these two proteins in carcinomas derived from different tissues (lung, esophagus, and breast) show similar quantities irrespective of the large differences found in their content in normal tissues (35). These findings support that during oncogenic transformation the tissue-specific differences in energy metabolism are abolished to converge on a similar phenotype to support tumor growth (35). In addition, the BEC index is a biomarker for cancer prognosis and response to therapy. In fact, a higher BEC index predicts a better overall survival and/or disease-free survival in acute myeloid leukemia patients and in colon, lung, breast, and ovarian cancer patients (36–42). These findings thus support that an impaired bioenergetic function of mitochondria favors recurrence and progression of the disease. Moreover, the BEC index also provides a tool for predicting the therapeutic response to various chemotherapeutic strategies aimed at combating tumor progression (43–46).

From a mechanistic view point the control of β-F1-ATPase expression is essentially exerted at post-transcriptional levels (47). In this regard, the translation of β-F1-ATPase mRNA (β-mRNA) both during development and in oncogenesis requires the specific activity of a *cis* element in the 3′ untranslated region of the mRNA that tightly controls its translation by RNA binding proteins (48–53) and miRNAs (54).

## The Diverse Role of Inhibitory Factor 1 (IF1) in Human Carcinomas

Besides the lower BEC index found in tumors, some prevalent human carcinomas also upregulate the expression of the ATPase IF1, which is the physiological inhibitor of the H^+^-ATP synthase (55, 56). Classically, IF1 was thought to function only to prevent mitochondrial ATP consumption by the reverse activity of the H^+^-ATP synthase (ATP hydrolase), which happens when mitochondria become de-energized such as in ischemia or in hypoxia (57, 58). However, more recent findings indicate that IF1 can bind to the H^+^-ATP synthase under normal phosphorylating conditions, hence inhibiting also the forward ATP synthetic activity of the enzyme (59). It should be noted that when arguing about the inhibition exerted by IF1 on the H^+^-ATP synthase it is important to take into consideration the tissue content of IF1 and the molar ratio that exists between IF1 and the H^+^-ATP synthase because the tissue availability of the inhibitor affects, among other factors, its interaction with the enzyme by the mass–action ratio. Unfortunately, the information of the tissue content of these two proteins in human and mouse tissues is presently missing.

In addition, it should be stressed that IF1 binding to the H^+^-ATP synthase, and hence its activity as an inhibitor of the enzyme, is subjected to a stringent posttranslational regulation of the protein by phosphorylation (59). In this regard, we have shown that IF1 is phosphorylated in S39 by a mitochondrial cAMP-dependent protein kinase that renders IF1 unable to bind to the H^+^-ATP synthase and hence inactive as an inhibitor of the enzyme (59). Regulation of IF1 phosphorylation depends on the cellular metabolic state to allow the fine tuning of ATP production to the cellular metabolic demand (56, 59). In this regard, we should stress that IF1 is found dephosphorylated, and hence active as an inhibitor of the enzyme in colon, lung, and breast carcinomas as well as in hypoxic cells and in cells progressing through the S/G2/M phases of the cell cycle (59).

In addition, IF1 is sharply upregulated in colon, lung, breast, and ovarian carcinomas, which are tissues that under normal physiological conditions are essentially devoid of the inhibitory protein (60–62). Not surprisingly, IF1 is an independent prognostic marker of disease progression for patients bearing these carcinomas. In non-small cell lung cancer (63), bladder carcinomas (64), and gliomas (65), a high expression level of IF1 in the tumor predicts a worse patient prognosis. On the contrary, in colon and breast cancer patients, a high level of IF1 expression predicts a better outcome (60, 66), especially in the bad prognosis group of triple-negative breast cancer patients (67). In the case of breast cancer, lymph node metastases show a lower expression level of IF1 when compared with the primary tumors (68). This finding suggested that breast cancer cells expressing low levels of IF1 may have a higher metastatic potential, which is in full agreement with our recent finding that low IF1 expression in triple-negative breast cancer cells confers a more invasive phenotype (67).

By contrast, human tissues that express high levels of IF1 under basal physiological conditions such as endometrium, kidney, liver, and stomach do not experience a relevant increase in IF1 expression by oncogenesis (60, 62). Nevertheless, in hepatocarcinomas (69) and in gastric carcinomas (70), a higher tumor content of IF1 predicts a worse prognosis for the patients. However, it should be mentioned that a higher IF1-mRNA expression level is correlated with a better prognosis in patients bearing the intestinal subtype of gastric cancer (66). This apparent discrepancy might arise from the histological type of gastric carcinomas analyzed in both studies and/or because the expression of IF1 in human carcinomas is primarily exerted at posttranscriptional levels (60). Overall, these findings support that IF1 plays a relevant role in cancer origin and progression. However, it remains to be elucidated the differential role played by IF1 in favoring or repressing cancer progression in different types of carcinomas, strongly emphasizing the need for specific studies in cellular, xenograft, and genetically modified mouse models in which to address these issues (67, 71).

## The Impact of IF1-Mediated Inhibition of the H^+^-ATP Synthase in Cancer

**Figure 2:**
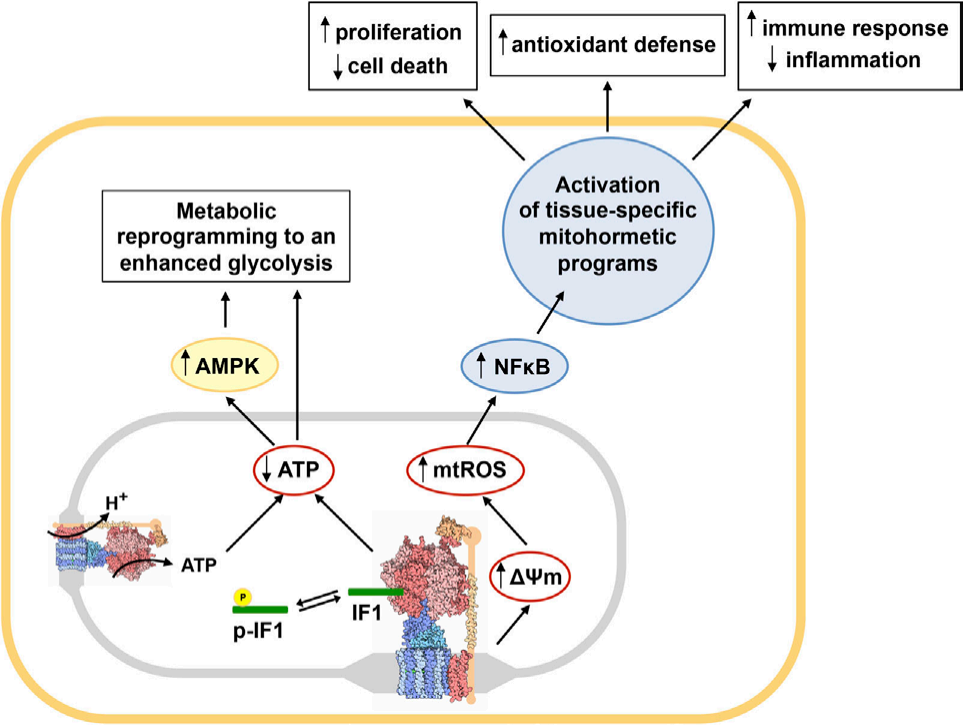
Main metabolic and redox circuits regulated by the inhibition of the H^+^-ATP synthase by inhibitory factor 1 (IF1). Dephosphorylated IF1 (green rod) inhibits the H^+^-ATP synthase (structure) when bound to the enzyme, while IF1 phosphorylation (yellow) prevents its binding and hence its inhibitory activity (59). The inhibition of a relevant pool of molecules of H^+^-ATP synthase by IF1 reduces cellular ATP availability, promoting metabolic reprogramming to an enhanced glycolysis and the activation of AMPK (71–73). Moreover, IF1 inhibition of the H^+^-ATP synthase triggers mitochondrial hyperpolarization because prevents H^+^ backflow through the enzyme enhancing the production of mitochondrial ROS (mtROS) that activate the canonical NFκB pathway (61, 72, 73). Activation of NFκB triggers the induction of different tissue-specific mitohormetic programs in the nucleus of the cell. In colon cancer cells overexpressing IF1, these programs favor proliferation, invasion, and resistance to cell death (61). In transgenic mice overexpressing IF1 in neurons or liver, promote the activation of survival pathways and antioxidant defenses (71, 72). In transgenic mice overexpressing IF1 in the intestine, the activated programs modulate the immune response of the tissue, favoring the development of an anti-inflammatory phenotype (73). Abbreviations: p-IF1, phosphorylated IF1; AMPK, AMP-activated protein kinase; NFκB, nuclear factor kappa B. The structure of the H^+^-ATP synthase was obtained from PDB.

The role of the H^+^-ATP synthase in cancer and in signaling has been studied by developing cellular and mouse models with regulated expression of IF1. The overexpression of IF1 both in cultured cells (55, 60, 61, 67) and in different tissues *in vivo* (71–73) is sufficient to promote metabolic reprogramming to an enhanced aerobic glycolysis (Figure 2). Upregulation of glycolysis results from the limitation of cellular ATP availability as a result of the inhibition of the H^+^-ATP synthase, supporting that the rate of ATP production by OXPHOS defines the rate of glucose consumption by aerobic glycolysis (74, 75). Likewise, this metabolic situation triggers the activation of the energy sensor AMPK (71–73) (Figure 2). Conversely, the silencing of IF1 in cells that express high levels of the protein has the opposite metabolic effect (55, 60, 61).

The inhibition of the H^+^-ATP synthase by IF1 also reduces the backflow of protons into the mitochondrial matrix, triggering mitochondrial hyperpolarization and a mild increase in the production of mitochondrial ROS (mtROS) (Figure 2), both *in vitro* and *in vivo* (55, 60, 61, 67, 72, 73). In cells and tissues overexpressing IF1 mtROS trigger the carbonylation of some cellular proteins and signal the activation of the canonical nuclear factor kappa B (NFκB) pathway (71–73) (Figure 2). In colon cancer cells, NFκB induces a nuclear transcriptional program that favors cellular proliferation, invasion, and evasion of cell death (61). These results are in line with other findings reporting that mtROS are necessary for proliferation and tumorigenesis and that ROS scavenging with mitochondrial-targeted antioxidants reduces cancer cell growth and prosurvival pathways (14, 76, 77). A recent study also argues that IF1 overexpression in carcinomas might contribute to cancer progression by limiting the processing of the pro-fusion dynamin-related protein optic atrophy 1 and thus limiting cristae remodeling during apoptosis (78).

However, the phenotypic changes triggered by IF1 overexpression in colon cancer cells cannot be generalized to other cellular types. In fact, the transcriptional program triggered by IF1 overexpression in triple-negative breast cancer cells supports just the opposite, a less proliferative and invasive phenotype (67). This phenotype for breast cancer cells was confirmed by functional analysis illustrating that IF1 overexpression promotes cell adhesion and maintenance of the extracellular matrix hampering epithelial to mesenchymal transition (67). Accordingly, the results may explain why breast cancer patients with high IF1 expression in the carcinoma have a better prognosis (60, 67).

In the mouse model overexpressing IF1-H49K (a constitutively active mutant of IF1) in the liver, we have shown that downregulation of OXPHOS triggers the induction of AMPK rendering a liver phenotype that is prone to cancer development (71). Indeed, transgenic mice when challenged with the carcinogen diethylnitrosamine develop more and bigger tumors than control mice because there is more extensive proliferation and diminished apoptosis of liver cells (71). Remarkably, IF1 overexpression in human hepatocarcinomas also triggers the activation of NFκB (Figure 2), which drives the promotion of angiogenesis and epithelial to mesenchymal transition (69). Not surprisingly, the expression of IF1 in liver cancer predicts a bad overall prognosis and the recurrence of the disease in these patients (69).

Mechanistically, although dimers of H^+^-ATP synthase are critical components of the PTP (19), and the overexpression of IF1 in the liver *in vivo* favors the formation of dimers of the enzyme (71), we have observed that cell death protection is not related to differential opening and regulation of the PTP (71). Actually, we support that the cell death protection afforded by IF1 overexpression in the liver is related to mitohormetic signaling through the induction of an antioxidant response guided by Nrf2 [nuclear factor (erythroid-derived 2)-like 2] (Figure 2) (71) because the metabolically preconditioned hepatocytes are more resistant to acetaminophen induced toxicity (71). Interestingly, Nrf2 upregulation is also a strategy deployed by cancer cells to detoxify the higher ROS levels that are produced in these cells (79).

## IF1-Mediated Inhibition of the H^+^-ATP Synthase Modulates Metabolic and Redox Circuits

Besides the liver, overexpression of IF1-H49K in neurons *in vivo* also reprograms energy metabolism to an enhanced glycolysis and affords metabolic preconditioning (72). In fact, mice overexpressing IF1-H49K in forebrain neurons are partially protected from excitotoxic damage induced by striatal administration of quinolinic acid because preconditioning partially protects neurons from death, reducing the lesion area in the brain and improving motor performance of the transgenic mice (72). Preconditioning in neurons also involves AMPK activation and mtROS-mediated signaling to implement a Bcl-xL-mediated protection of neurons from apoptosis (72) (Figure 2). Other findings also support the neuroprotective role of IF1 in ischemia reoxygenation by promoting autophagy and maintaining mitochondrial bioenergetics (80), although the contribution of IF1 to promotion of autophagy in neurons remains to be studied in the *in vivo* model.

Interestingly, the signaling pathways triggered by the IF1-mediated inhibition of the H^+^-ATP synthase are not limited to the cells overexpressing IF1 but also implicate non-cell autonomous processes. The transgenic mice overexpressing IF1 in enterocytes also show metabolic reprogramming to an enhanced glycolysis and activation of mtROS–NFκB signaling pathway (73) (Figure 2). Transgenic mice are partially protected from intestinal inflammation after the administration of the inflammatory agent dextran sodium sulfate (DSS), due to increased recruitment of regulatory T cells and macrophages that are mainly polarized to the M2 phenotype (73). These findings are consistent with the anti-inflammatory phenotype afforded by the overexpression of IF1 as revealed by the higher levels of anti-inflammatory cytokines present in plasma and intestine of the transgenic mice (73). Colonocytes of control mice induce the oncogenic Akt/mTOR/p70S6K and pro-inflammatory STAT3 pathways upon administration of DSS, something that is not observed in IF1-transgenic mice (73). Preconditioning and protection against stress in colon of IF1-transgenic mice clearly responds to the basal activation of the NFκB pathway due to mtROS production (73) (Figure 2), because protection from intestinal inflammation is blunted when an mtROS scavenger or an inhibitor of NFκB are administered (73).

Overall, transgenic mice overexpressing IF1 in liver, brain, or intestine reveal that by partial inhibition of OXPHOS and the production of mtROS the tissues acquire an advantageous phenotype against different forms of oxidative stress and inflammation (71–73) (Figure 2), stressing the role of the H^+^-ATP synthase as a therapeutic target in diverse human pathologies.

## Longevity and the Inhibition of the H^+^-ATP Synthase

Besides IF1, the H^+^-ATP synthase can be inhibited by some mitochondrial metabolites produced in the TCA cycle, such as α-ketoglutarate (α-KG) (81). Therefore, partial inhibition of the enzyme by α-KG also reduces ATP availability and TOR signaling, promoting autophagy in *Caenorhabditis elegans* (81). In addition, the oncometabolite (R)-2-hydroxyglutarate (2-HG), which is structurally similar to α-KG, also inhibits the H^+^-ATP synthase both in *C. elegans* and in mammalian cell lines (82). 2-HG is highly accumulated in some gliomas and acute myeloid leukemias that harbor mutations in the genes encoding the cytosolic and mitochondrial isocitrate dehydrogenases (IDH1 and IDH2, respectively). These mutations result in neomorphic enzymes with higher affinity for α-KG that catalyze its conversion into 2-HG (83, 84). Interestingly, the inhibition of the H^+^-ATP synthase by 2-HG or α-KG in glioblastoma cells triggers cell growth arrest and cell death under conditions of limited glucose (82). These results are consistent with previous findings that indicate that brain cancer patients with IDH mutations have a longer median overall survival than patients without mutations (83, 85). The suppression of cell growth may be ascribed to reduced ATP levels and mTOR signaling in the tumors (82).

Interestingly, both α-KG and 2-HG, by inhibiting the H^+^-ATP synthase, also extend lifespan in *C. elegans* (81, 82), and α-KG might play a role in longevity induced by dietary restriction (81). Other interventions targeting the H^+^-ATP synthase have also been shown to extend lifespan in several model organisms, as recently reviewed (62). For instance, silencing of subunits of the H^+^-ATP synthase in *C. elegans* and *Drosophila melanogaster* promotes longevity (86, 87). No contribution to longevity has been reported so far in mammals for the modulation of the H^+^-ATP synthase. However, recent findings indicate that the specific inhibition of the enzyme by the small molecule J147 prevents the age-associated drift of the hippocampal transcriptome and plasma metabolome in mice and extends lifespan in *D. melanogaster*, providing an additional link between the activity of the H^+^-ATP synthase, aging and age-associated pathologies such as dementia (88).

Overall, these findings support the notion that the H^+^-ATP synthase is also a conserved hub in intracellular signaling that plays a key role in signaling mitohormesis contributing to cell fate decisions and longevity.

## Concluding Remarks

Reprogramming cellular metabolism is a hallmark of cancer that is necessary to fulfill the metabolic demands imposed by the oncogenic process. In this context, the mitochondrial H^+^-ATP synthase is a main hub in rewiring energy metabolism and in retrograde signaling to the nucleus programs required for cancer progression. In this regard, most prevalent carcinomas show reduced expression of the catalytic subunit of the H^+^-ATP synthase (β-F1-ATPase) relative to the glycolytic GAPDH, what provides a protein signature of energy metabolism of clinical relevance in oncogenesis. Moreover, some prevalent carcinomas also show an increased expression of IF1, the physiological inhibitor of the enzyme. As revealed in different *in vitro* and *in vivo* systems, IF1 overexpression is sufficient to rewire energy metabolism to an enhanced glycolysis and to trigger an mtROS signal that promotes nuclear reprogramming. IF1-mediated reprogramming is mainly geared by the activation of AMPK and NFκB pathways resulting in the induction of tissue-specific programs aimed at preventing cell death, oxidative damage or inflammation. The precise molecular events that lead to the upregulation of IF1 in cancer and its role in cancer progression in different carcinomas remain to be established. The H^+^-ATP synthase, engine of OXPHOS, is also a crucial hub in mitohormetic signaling to modulate cytoprotective defenses that contribute to longevity in several organisms. Therefore, deciphering the metabolic and redox circuits controlled by the H^+^-ATP synthase and IF1 are of utmost importance to understand how they contribute to oncogenesis and thus providing new targets for cancer and age-associated diseases.

## Author Contributions

PE-M and JC wrote the paper. All the authors read, contributed, and approved the final manuscript.

## Conflict of Interest Statement

The authors declare that the research was conducted in the absence of any commercial or financial relationships that could be construed as a potential conflict of interest.
